# Cerebral Hemodynamics During a Cognitive-Motor Task Using the Limbs

**DOI:** 10.3389/fnhum.2020.568030

**Published:** 2020-11-10

**Authors:** Akira Sagari, Hiroyo Kanao, Hitoshi Mutai, Jun Iwanami, Masaaki Sato, Masayoshi Kobayashi

**Affiliations:** ^1^Division of Occupational Therapy School of Health Science, Faculty of Medicine, Shinshu University, Matsumoto, Japan; ^2^Rehabilitation Division, Kami-iida Rehabilitation Hospital, Nagoya, Japan

**Keywords:** near-infrared spectroscopy, oxygenated hemoglobin, antagonistic task, cerebral hemodynamics, visual analog scale

## Abstract

**Background**: Antagonistic tasks are cognitive-motor task trainings. Intervention programs involving antagonistic exercise tasks are being employed to help prevent falls and reduce the need for nursing care in older populations. Meanwhile, the effects of such tasks on blood flow in the brain remain obscure. This study aimed to clarify the effects of antagonistic tasks on prefrontal cortical cerebral hemodynamics.

**Materials and Methods**: We assessed 13 healthy adults (two men, 11 women; mean age, 21.4 ± 1.0 years). Participants imitated each of the antagonistic tasks presented on a PC monitor placed at a 120-mm viewing distance. All participants performed six tasks, consisting of upper-limb tasks (non-antagonism, simple antagonism, and complex antagonism) and upper- and lower-limb tasks (tasks combining lower-limb opening and closing movements with each upper-limb task). We used near-infrared spectroscopy (NIRS) to measure cerebral blood flow dynamics, with oxygenated hemoglobin (Oxy-Hb) concentration changes as the main outcome. A 10-channel probe was placed on the participants’ forehead, focusing on the prefrontal cortex. We first obtained a baseline NIRS measurement for 10 s; the participants then imitated the task presented on the PC monitor for 90 s. We measured the number of errors and the subjective difficulty of each task.

**Results**: The increase in prefrontal cortex Oxy-Hb concentration was significantly higher in the complex antagonist conditions than in the non-antagonistic and simple antagonistic conditions. There were no significant prefrontal cortex Oxy-Hb differences between the upper limb and upper- and lower-limb conditions (increasing number of motor limbs).

**Conclusions**: The study findings support that an increase in finger-shaped complexity has a greater effect on cerebral blood flow dynamics in the prefrontal cortex than does an increase in the number of motor limbs involved in the task.

## Introduction

Over 28% of individuals in Japan are aged 65 years or older, and the number of adults over 75 years is increasing (Cabinet Office Government of Japan, 2019). For many years, several care prevention projects have been implemented in various settings to promote healthy lifestyles among older adults in Japan. Numerous interventions combine cognitive and exercise tasks in the context of care prevention programs for community-dwelling older adults (Shigematsu et al., 2008a,b; Suzuki et al., 2013). These interventions seek to prevent falls and activate cognitive functions, effectively reducing the need for nursing care (Pichierri et al., 2011; Kojima et al., 2017). These interventions are designed to impose a dual-task and cognitive load on the participants. Antagonistic exercise is a program frequently implemented by occupational therapists and care workers (Tabira et al., 2012). They consist of a task in which the patients perform opposing movements using their left and right upper limbs and their upper and lower limbs in a rhythmic manner. Antagonistic exercise helps improve attention and working memory. The purpose of this exercise focuses on maintaining and improving abilities in older adults such as attention and working memory. Despite its implementation in many medical and nursing facilities, the selection of tasks is based on empirical rules, for which the difficulty and effects of the antagonistic manipulation techniques remain unknown.

Previously, we implemented a program that included antagonistic exercises for older adults in the community and observed improvements in memory and attention functions for 6 months (Sagari et al., 2012). However, the effects of antagonistic exercise on cognitive function were difficult to assess due to the complexity of the intervention program. Recently, near-infrared spectroscopy (NIRS) has been frequently used as a means of capturing cerebral blood flow dynamics during exercise and cognitive tasks, allowing researchers to assess the effects of such tasks on humans. Numerous studies have examined cerebral hemodynamics using NIRS during a wide variety of motor activities, such as running or walking (Suzuki et al., 2004; Harada et al., 2009), cycling (Ide et al., 1999), and finger tapping (Holper et al., 2009). Moreover, researchers have reported cerebral hemodynamics during cognitive tasks, such as trail building (Ohsugi et al., 2013), rock-paper-scissors (Yamauchi et al., 2013), motor imagery (Iso et al., 2016), and sequential finger touching (Amemiya et al., 2010; Sagari et al., 2015). In a prior study, we used NIRS to examine the effects of antagonistic exercise tasks of varying degrees of difficulty on cerebral hemodynamics in the prefrontal cortex and observed an increase in oxygenated hemoglobin (Oxy-Hb) values in the bilateral prefrontal cortex (Tabira et al., 2012) as the task complexity increased. However, since the task consisted solely of hand movements, it did not reflect the antagonistic manipulation-based interventions performed in medical and nursing care facilities, which engages both the upper and lower limbs, with flexing and extending exercises of the elbow joints. In addition, in the previous study, we only examined the four asymmetrically located NIRS probes, and there was a methodological problem. Therefore, in this study, we analyzed all the NIRS probes that could measure our data. We originally planned to conduct this study on the elders; however, it was easier to target university students, therefore the subjects are younger adults. The objective of our study was to clarify the effects of several characteristics of antagonistic tasks on prefrontal cortical cerebral hemodynamics. According to our hypothesis, the prefrontal cortex is more activated in the upper and lower-limb motor tasks than in upper-limb tasks alone. In addition, we predicted that activation of the prefrontal cortex may also occur due to finger-shape complexity.

## Materials and Methods

### Participants and Experimental Procedures

In this study, we assessed 13 healthy adults (two men, 11 women; mean ± standard deviation age, 21.4 ± 1.0 years) between September 2017 and March 2020. Participant eligibility included age >20 years and the ability to perform normal exercises. Individuals with a history of central nervous system disorders were excluded.

**Figure 1:**
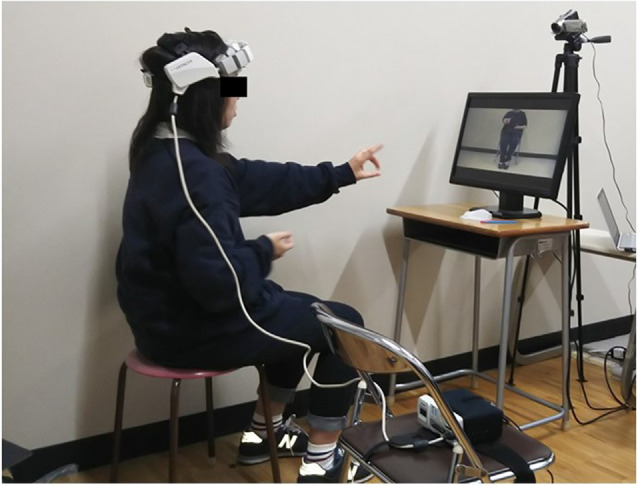
Experimental setup of the antagonistic exercise tasks. The photograph shows a subject wearing a headset probe with the near-infrared spectroscopy (NIRS) device. The tasks being performed were recorded by the video camera. The participants had to imitate various tasks presented on a PC monitor placed in front of the subject at a viewing distance of 120 mm.

**Figure 2:**
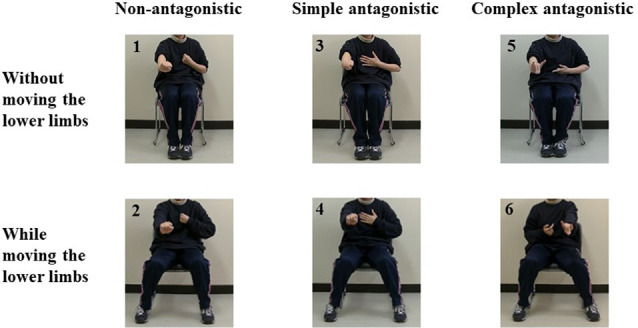
Pictures depicting the antagonistic exercise tasks used in this study. Task 1: Non-antagonistic task using the upper limbs. Task 2: Non-antagonistic task using the upper and lower limbs. Task 3: Simple antagonistic task using the upper limbs. Task 4: Simple antagonistic task using the upper and lower limbs. Task 5: Complex antagonistic task using the upper limbs. Task 6: Complex antagonistic task using the upper and lower limbs.

The participants imitated each of the antagonistic tasks presented on a PC monitor (510 × 210 mm) placed at a 120-mm viewing distance as they remained seated in a calm environment (Figure 1). They performed six tasks: tasks involving the upper limbs only (tasks involving non-antagonism, simple antagonism, and complex antagonism) and those involving both the upper and lower limbs (wherein lower-limb opening and closing movements were performed simultaneously with each upper-limb task; Figure 2). NIRS (WOT-100: HITACHI, Tokyo, Japan; Leaflets of WOT-100, 2020) was used to measure cerebral blood flow dynamics. The 10-channel probe was placed on the participants’ forehead, focusing on the prefrontal cortex (Atsumori et al., 2009). Thirty seconds following an instruction to close their eyes, the participants were instructed to reopen them and look at the cross located at the center of the PC monitor for 10 s. This 10 s was used as the baseline for NIRS measurement. Subsequently, participants imitated the task presented on the PC monitor for 90 s. We previously prepared randomized task sheets and allocated them to the subjects. The task was recorded by a video camera (GZ-E242-S Everio, JVC, Yokohama, Kangawa, Japan) to determine the number of errors. This number was counted during the task and later confirmed by repeated observation of the recorded video by an inspector. An error was considered for clearly incorrect imitated movements. For evaluation of the subjective difficultly of each task, participants were given a visual analog scale (VAS; Wewers and Lowe, 1990) following completion of all tasks and instructed to rate the difficulty of the task using the scale.

This study was approved by the ethical review board of Shinshu University School of Medicine (Study No. 3818). All procedures were carried out in accordance with the ethical standards of the Declaration of Helsinki. All participants provided written informed consent.

### NIRS Measurements

**Figure 3:**
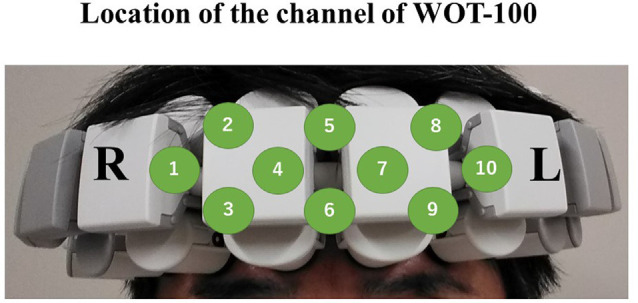
Schematic of the probe unit, covering an area of 30 × 105 mm^2^ on the participant’s foreheads including both temples. This arrangement enabled the monitoring of cortical activation, mainly in the prefrontal cortex. All channels are labeled from 1 to 10.

NIRS measurements were performed using a continuous-wave system equipped with 2 × 4 optode probe sets (eight incident light and 10 detector fibers), resulting in a total of 10 channels with an inter-optode distance of 30 mm. The probe unit covered an area of 30 × 105 mm^2^ on the participants’ foreheads, including both temples. This arrangement enabled the monitoring of cortical activation, mainly in the prefrontal cortex (Atsumori et al., 2009). The prefrontal cortex is responsible for attention and working memory (Carlen, 2017). The probe sets are shown in Figure 3. The continuous-wave NIRS system uses two different wavelengths (~790 and 850 nm). Relative changes in the absorption of near-infrared light were sampled at 5 Hz, converting them into related concentration changes for Oxy-Hb and deoxygenated hemoglobin based on the modified Beer–Lambert approach (Obrig and Villringer, 2003). The moving average method (with a 5 s window) was used to exclude any short-term motion artifacts in the analyzed data. Baseline was defined as the 10-s period prior to task onset. We also calculated the average value for each of the channel data during the 90-s task performance period, and then these values were averaged over 10 channels. Additionally, we used changes in Oxy-Hb concentration as an indicator of fluctuations in the regional cerebral blood volume, since an earlier NIRS signal study using a perfused rat brain model proposed that Oxy-Hb, rather than deoxygenated hemoglobin, is the most sensitive parameter for an activation study. Oxy-Hb is an indicator of local neural activity rather than an indicator of fluctuations in regional cerebral blood volume (Hoshi et al., 2001).

### The Antagonistic Task

In Task 1 (non-antagonistic task using the upper limbs; NU), the elbows were alternately bent and stretched once every 2 s approximately, with all fingers of both hands fully flexed to form a fist. For Task 2 (non-antagonistic task using the upper and lower limbs; NUL), the lower limbs were alternately opened and closed once every 2 s approximately while performing Task 1. In Task 3 (simple antagonistic task using the upper limbs; SU), the left elbow was placed in a flexed position and the fingers of the left hand were in full flexion to form a fist, while the right elbow was placed in an extended position and the fingers of the right hand were in full extension (i.e., in the “paper” shape from rock-paper-scissors). The positions were alternated between the right and left hands according to the set rhythm. In Task 4 (simple antagonistic task using the upper and lower limbs; SUL), the lower limbs were alternately opened and closed approximately once every 2 s while performing Task 3. In Task 5 (complex antagonistic task using the upper limbs; CU), the right thumb and index finger were extended; the middle, ring, and little fingers were flexed (“gun” shape); and the elbow was flexed. On the left hand, the thumb, middle finger, and ring finger were flexed; the index finger and little finger were extended (“wolf head” shape); and the elbow was alternately extended and bent. The positions were alternated between the right and left hands according to the set rhythm approximately once every 2 s. In Task 6 (complex antagonistic task using the upper and lower limbs; CUL), the lower limbs were alternately opened and closed approximately once every 2 s during Task 5.

### Statistical Analysis

Normality assumption was verified using the Shapiro–Wilk test. We compared Oxy-Hb values across the six tasks using one-way repeated measures analysis of variance (ANOVA), with Bonferroni tests for multiple comparisons. We compared the subjective difficulty and number of errors across the six tasks using Friedman’s test and Scheffe tests for multiple comparisons. Additionally, the number of errors, subjective difficulty, and Oxy-Hb values were divided according to the following factors: upper limbs/upper limbs and lower limbs (increasing number of motor limbs), and non-antagonism/simple antagonism/complex antagonism (increasing finger-shape complexity). For Oxy-Hb comparisons, a two-way repeated-measures ANOVA was employed with the significance level set at *p* < 0.05. For subjective difficulty and number of error comparisons, Friedman’s test with Scheffe tests was used with the significance level set at *p* < 0.05. The correlation coefficients between Oxy-Hb and VAS were analyzed by calculating Spearman’s rank correlation coefficients. The effect size was calculated for the test in Oxy-Hb only. Statistical analyses were performed using the BellCurve for Excel (Social Survey Research Information Co., Limited, Tokyo, Japan).

## Results

Oxy-Hb data followed a normal distribution, while VAS scores and number of errors did not. The results (numerical values) for VAS scores, number of errors, and Oxy-Hb concentration changes are presented in Table 1. With regard to subjective difficulty, Friedman’s test revealed a significant main effect of subjective difficulty among tasks (*p* < 0.001). *Post hoc* tests showed a significant increase in subjective difficulty for SUL, CU, and CUL compared to the subjective difficulty of NU (*p* = 0.037, *p* < 0.001, *p* < 0.001). In addition, *post hoc* tests showed a significant increase in subjective difficulty for CU and CUL when compared to that of SU (*p* = 0.024, *p* < 0.001) and for NUL vs. CU (*p* = 0.028) and CUL vs. NUL (*p* = 0.001). Friedman’s test revealed a main effect between the upper limb and upper- and lower-limb conditions (increasing number of motor limbs) in terms of subjective difficulty (VAS scores; *p* < 0.001). *Post hoc* tests showed a significant increase in subjective difficulty for tasks involving both the upper and lower limbs compared to tasks involving only the upper limbs (*p* < 0.001). The main effect of subjective difficulty was also observed between non-antagonistic, simple antagonistic, and complex antagonistic conditions (increasing finger-shape complexity; *p* < 0.001). *Post hoc* tests revealed that the complex antagonistic conditions were significantly more challenging than the non-antagonistic and simple antagonistic conditions (*p* < 0.001, *p* < 0.001).

**Table 1:** Subjective difficulty VAS scores, number of errors, and Oxy-Hb concentration changes in the prefrontal cortex for each task.

	Non-antagonistic task using the upper limbs	Non-antagonistic task using the upper and lower limbs	Simple antagonistic task using the upper limbs	Simple antagonistic task using the upper and lower limbs	Complex antagonistic task using the upper limbs	Complex antagonistic task using the upper and lower limbs
VAS score, mm	4 (1–3)	15 8–20)	13 (2–27)	28 (10–38)	61 (47–68)	80 (51–86)
Number of errors	0 (0–0)	0 (0–0)	0 (0–0)	0 (0–1)	3 (0–23)	1 (0–16)
Prefrontal cortex Oxy-Hb concentration changes, mMmm	0.02 ± 0.04	0.06 ± 0.06	0.01 ± 0.04	0.01 ± 0.05	0.18 ± 0.07	0.15 ± 0.08

*Note. Data of VAS score and number of errors are presented as the median and interquartile range. Data of prefrontal cortex Oxy-Hb concentration changes are presented as the mean ± standard deviation. VAS, visual analog scale; Oxy-Hb, oxygenated hemoglobin*.

Friedman’s test showed a significant main effect of the number of errors in each task (*p* < 0.001). *Post hoc* tests revealed a significantly higher number of errors in CU than in NU (*p* = 0.026) and in CU than in SU (*p* = 0.018) or in NUL than in CU (*p* = 0.018). Friedman’s test showed no main effect between the upper limb and upper- and lower-limb conditions (increasing number of motor limbs) in terms of error count. The main effect of the number of errors was observed among non-antagonistic, simple antagonistic, and complex antagonistic conditions (increasing finger-shape complexity; *p* < 0.001). *Post hoc* tests revealed that the number of errors was significantly higher in the complex antagonistic than in the non-antagonistic and simple antagonistic conditions (*p* < 0.001, *p* = 0.007).

**Figure 4:**
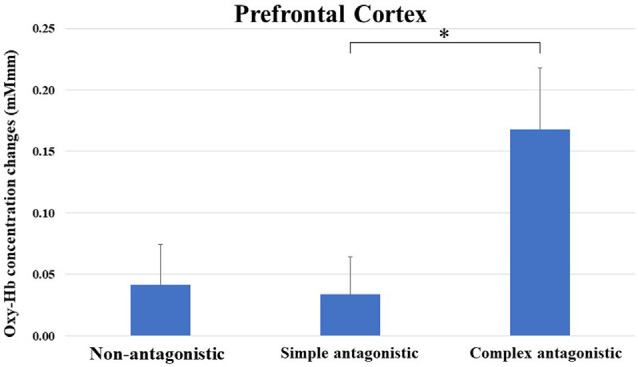
Oxy-Hb concentration changes in the prefrontal cortex during non-antagonistic, simple antagonistic, and complex antagonistic tasks. Two-way repeated measures analysis of variance (ANOVA) and *post hoc* tests revealed a significant difference in Oxy-Hb levels between simple and complex antagonistic tasks. *p < 0.05. Oxy-Hb, oxygenated hemeoglobin.

**Figure 5:**
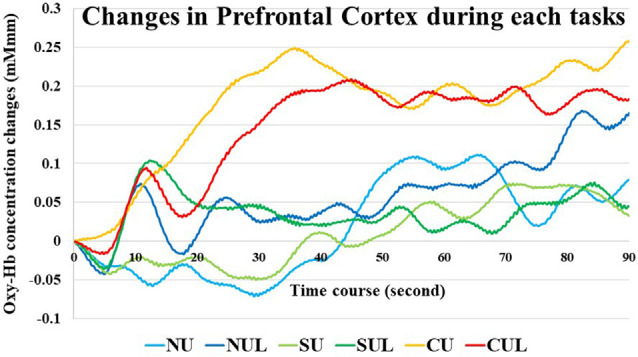
Time courses of Oxy-Hb concentration changes in the prefrontal cortex during each task. NU, non-antagonistic task using the upper limbs; NUL, non-antagonistic task using the upper and lower limbs; SU, simple antagonistic task using the upper limbs; SUL, simple antagonistic task the upper and lower limbs; CU, complex antagonistic task using the upper limbs; CUL, complex antagonistic task using the upper and lower limbs.

**Figure 6:**
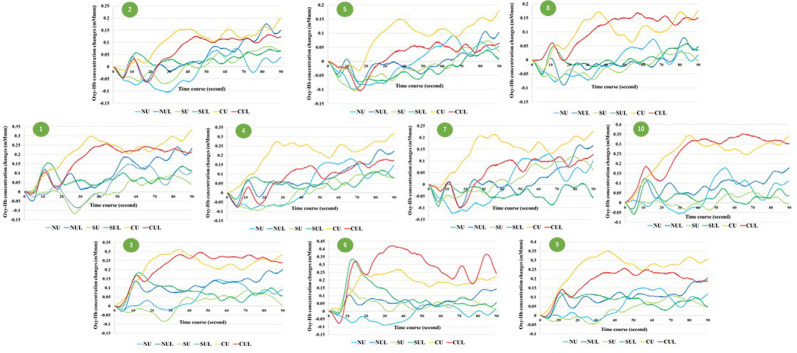
Time courses of Oxy-Hb concentration changes in all channel. NU, non-antagonistic task using the upper limbs; NUL, non-antagonistic task using the upper and lower limbs; SU, simple antagonistic task using the upper limbs; SUL, simple antagonistic task using the upper and lower limbs; CU, complex antagonistic task using the upper limbs; CUL, complex antagonistic task using the upper and lower limbs.

Oxy-Hb concentration changes across tasks did not show a significant effect in a one-way repeated measures ANOVA, and a two-way repeated-measures ANOVA did not identify a significant effect between the upper limb and upper- and lower-limb conditions (increasing number of motor limbs). The main effect of Oxy-Hb concentration was observed among non-antagonistic, simple antagonistic, and complex antagonistic conditions (increasing finger-shape complexity; *p* = 0.02; η^2^ = 0.09). The *post hoc* test revealed significantly higher values for the complex antagonistic condition than for the non-antagonistic and simple antagonistic conditions (*p* = 0.017, *p* = 0.024; *r* = 0.40, *r* = 0.51; Figure 4). There was a significant positive correlation between Oxy-Hb and VAS (ρ = 0.344, *p* = 0.002). Time courses of Oxy-Hb concentration changes in the prefrontal cortex during each task are shown in Figures 5, 6.

## Discussion

### Subjective Difficulty and Errors in the Antagonistic Task

With regard to subjective difficulty (VAS score), NU was the least challenging, while CUL was the most difficult. The other tasks showed increasing difficulty in the following order: NUL, SU, SUL, and CU. Similarly, the tasks were associated with an increasing number of errors in the following order: NUL (minimum errors), SU, NU, SUL, CU, and CUL (maximum errors). Also, increasing finger-shape complexity increased both subjective difficulty and number of errors. This trend was particularly significant in the complex antagonistic task. A previous study using an antagonistic task (Tabira et al., 2012) demonstrated an increased number of errors as the hand shape became increasingly complex. Therefore, the results of the current study were similar to those of the previous study. Adding the lower-limb opening and closing motion to the upper-limb task increased the subjective difficulty, although it is insufficient to induce an error. Therefore, increasing finger-shape complexity and an increasing number of motor limbs could increase subjective difficulty in the antagonistic task.

### Cerebral Blood Flow Dynamics During an Antagonistic Task

The prefrontal cortex Oxy-Hb did not significantly differ between the upper limb and upper- and lower-limb conditions (increasing number of motor limbs). However, a significant difference was found between the non-antagonistic, simple antagonistic, and complex antagonistic conditions (increasing finger-shape complexity). Additionally, the complex antagonistic condition increased the Oxy-Hb concentration in the prefrontal cortex, significantly more than did the non-antagonistic and simple antagonistic conditions. This phenomenon could result from subjective difficulty induced by the complex antagonistic condition. As a result, we speculated that errors would increase under the complex antagonistic condition. In a previous study (Tabira et al., 2012), the Oxy-Hb concentration in the prefrontal cortex increased with the difficulty of the antagonistic task in older adults. This finding suggested that the prefrontal cortex was activated by increasingly complex finger shapes. The results of the previous study are similar to our current results. However, the previous study did not include the lower-limb condition. In this study, we found that the prefrontal cortex was not activated by the increasing number of motor limbs, e.g., the inclusion of the lower limbs. However, the current study’s subjects consisted of young adults. This highlights the differences in the prefrontal cortical hemodynamic response to the task between the young and old adults (Ohsugi et al., 2013; Beurskens et al., 2014). Therefore, the results of this study should be interpreted with caution. Previously, some studies investigated cerebral blood flow in the prefrontal cortex during cognitive-motor tasks (Holtzer et al., 2011, 2017; Mirelman et al., 2014). These studies reported that by adding a computational task to gait and adjusting difficulty, the prefrontal cortex was activated when the task became difficult. This task was a very rare one, and difficulty adjusted according to the complexity of the movement. Even in such a task, it became clear that the prefrontal cortex was activated as the task became more difficult. In addition, in the prefrontal cortex Oxy-Hb increased rapidly or slowly during the task and was maintained in the final stages as the test subjects had to concentrate on the task while doing it. However, if the participants become accustomed to the task, the changes in Oxy-Hb could decrease over time (Sagari et al., 2012; Alves Heinze et al., 2019).

### Clinical Application

Antagonistic exercise is widely used in medical and nursing care facilities for cognitive function improvement and maintenance (Nagasaki Prefectural Government, Community Support Activity, 2020; Tokushukai Medical Group Newspaper Digest, 2020). According to this study’s results, the complex antagonistic task was effective in activating the prefrontal cortex. Half of the participants made several errors during the complex antagonistic task, suggesting that the tasks responsible for occasional incorrect movements are effective at stimulating brain activity. Additionally, the subjective difficulty experienced by participants during the complex antagonistic task was approximately 60–80 mm, which might help predict the related brain activity. Using NIRS, cognitive function improvement and maintenance programs can be enhanced while providing feedback on the prefrontal activation status (Mihara et al., 2013; Kinoshita et al., 2016). However, in actual medical and nursing care facilities, it is more realistic to select tasks based on the predicted activation status of the prefrontal cortex, which can be determined by referring to the VAS scores and error counts associated with a task.

### Limitations

This study had several limitations. First, the number of errors was counted by an inspector. Since the data are subjective, the development of devices to record objective data, without user bias, is required. Second, the lower-limb task used in this study consisted of lower-limb opening and closing, and simple movements might not have made a difference in the cerebral hemodynamics when comparing the upper limb and the upper- and lower-limb conditions (increasing number of motor limbs). Further complexity of the lower-limb task may result in differences in the cerebral hemodynamics of the prefrontal cortex. Third, activation in the supplementary motor area, dorsal premotor cortex, and sensorimotor cortex, which are linked to the prefrontal cortex, could not be detected due to the technical limitations of the NIRS apparatus (WOT-100), which has a relatively small probe. Fourth, since the study participants were young adults, future studies with older adults are warranted. Using G power, assuming *α* = 0.05 (1-β) = 0.8 and effect size = 0.25, 24 patients were required for our study. Unfortunately, we were unable to obtain this target number and the male–female ratio was greatly skewed. Finally, because of the differences in the male-to-female ratio in the cohort, future studies should include more male subjects.

## Conclusion

The purpose of this study was to clarify the effects of antagonistic tasks on prefrontal cortical cerebral hemodynamics. Our study findings showed that the complex antagonistic condition increased Oxy-Hb in the prefrontal cortex, more than the non-antagonistic and simple antagonistic conditions. These findings indicate that areas of the subjects’ prefrontal cortex were activated by increasingly complex finger shapes during the antagonistic task. These results support that an increase in the number of motor limbs involved in the task has fewer effects on the cerebral blood flow dynamics in the prefrontal cortex than does an increase in finger shaped complexity. This information could be helpful for occupational therapists when recommending antagonistic exercise.

## Data Availability Statement

The raw data supporting the conclusions of this article will be made available by the authors, without undue reservation.

## Ethics Statement

The studies involving human participants were reviewed and approved by the ethical review board of Shinshu University School of Medicine (Study No. 3818). The patients/participants provided their written informed consent to participate in this study.

## Author Contributions

Contributions were as follows: AS, HM, and MK: study concept and design. AS, HK, MS, and JI: data acquisition. AS, HK, and HM: data analysis and interpretation. AS and MK: manuscript writing. All authors contributed to the article and approved the submitted version.

## Conflict of Interest

The authors declare that the research was conducted in the absence of any commercial or financial relationships that could be construed as a potential conflict of interest.
